# Preference Transitivity and Symbolic Representation in Capuchin Monkeys (*Cebus apella*)

**DOI:** 10.1371/journal.pone.0002414

**Published:** 2008-06-11

**Authors:** Elsa Addessi, Alessandra Mancini, Lara Crescimbene, Camillo Padoa-Schioppa, Elisabetta Visalberghi

**Affiliations:** 1 Unit of Cognitive Primatology and Primate Center, Institute of Cognitive Sciences and Technologies, Consiglio Nazionale delle Ricerche (CNR), Rome, Italy; 2 University “La Sapienza”, Rome, Italy; 3 Department of Neurobiology, Harvard Medical School, Boston, Massachusetts, United States of America; 4 Washington University School of Medicine, St Louis, Missouri, United States of America; Yale University, United States of America

## Abstract

**Background:**

Can non-human animals comprehend and employ symbols? The most convincing empirical evidence comes from language-trained apes, but little is known about this ability in monkeys. Tokens can be regarded as symbols since they are inherently non-valuable objects that acquire an arbitrarily assigned value upon exchange with an experimenter. Recent evidence suggested that capuchin monkeys, which diverged from the human lineage 35 million years ago, can estimate, represent and combine token quantities. A fundamental and open question is whether monkeys can reason about symbols in ways similar to how they reason about real objects.

**Methodology/Principal Findings:**

Here we examined this broad question in the context of economic choice behavior. Specifically, we assessed whether, in a symbolic context, capuchins' preferences satisfy transitivity - a fundamental trait of rational decision-making. Given three options A, B and C, transitivity holds true if A≥B, B≥C and A≥C (where ≥ indicates preference). In this study, we trained monkeys to exchange three types of tokens for three different foods. We then compared choices monkeys made between different types of tokens with choices monkeys made between the foods. Qualitatively, capuchins' preferences revealed by the way of tokens were similar to those measured with the actual foods. In particular, when choosing between tokens, monkeys displayed strict economic preferences and their choices satisfied transitivity. Quantitatively, however, values measured by the way of tokens differed systematically from those measured with the actual foods. In particular, for any pair of foods, the relative value of the preferred food increased when monkeys chose between the corresponding tokens.

**Conclusions/Significance:**

These results indicate that indeed capuchins are capable of treating tokens as symbols. However, as they do so, capuchins experience the cognitive burdens imposed by symbolic representation.

## INTRODUCTION

Humans have been defined “the symbolic species” since the use and understanding of symbols drastically transformed our hominid ancestors throughout evolution [1]. The acquisition of a complex language is unparalleled in the animal realm and probably underlies human uniqueness [2]. Besides language, humans creatively and flexibly use a huge array of symbols, thus acquiring information about the world without having direct experience of all its features. The use of symbols makes it possible to travel both in time and space and to accumulate and transmit cultural knowledge over generations [3]–[5].

Whether non-human animals comprehend and employ symbols is still an open question since symbolic competence is difficult to test in the absence of language. The most convincing empirical evidence of animals using symbols comes from a series of studies on language-trained apes. Two chimpanzees learned to use lexigrams to ask one another's for the appropriate tool required to obtain food and they readily fulfill to one another's requests [6]. Moreover, chimpanzees trained to sort out real foods from real tools and to categorize each of them by choosing the consistent lexigram out of two (generically indicating one ‘food’ and the other ‘tool’) kept categorizing using the correct lexigram also when presented with new items [7], [8]. Furthermore, in a reverse-reward contingency task [9]–[11], where chimpanzees failed to select a smaller food array in order to receive a larger one, the use of Arabic numerals (instead of food) allowed chimpanzees to overcome their strong motivation to choose the largest between the two food arrays and to be successful.

Little is known about the symbolic ability of non-apes. There is some evidence that capuchin monkeys, South-American primates that diverged from us 35 million years ago, use tokens as symbols [12], [13]. Tokens are inherently non-valuable objects that acquire an associative value upon exchange with the experimenter [14]. Following DeLoache's [5] definition of symbol (“something that someone intends to represent something other than itself”, p.66), a token can be considered a symbol since it is arbitrarily related to its referent through the conventions established between the experimenter and the exchanging subject [15], [16].

Numerous studies in recent years examined aspects of economic behavior in non-human primates using tokens. For example, tokens were used to test reactions to social inequity [17, but see 18,19], reference-dependent preferences [20], and endowment effects [21]. In all these experiments, monkeys were typically asked to trade with the experimenter valueless objects (the tokens) in exchange for desirable pieces of food. Probing economic preferences using tokens opens a number of important questions. For example, monkeys could psychologically treat tokens as symbols for the food they represent, similarly to how humans treat words or money. Alternatively, exchanging tokens for food could simply result from instrumental conditioning. In this scenario, monkeys exchanging tokens with the experimenters would display a behavior conceptually analogous to the behavior of pigeons operating a lever to obtain food.

A closely related question is whether preferences monkeys reveal by the way of tokens are qualitatively and quantitatively similar to those they reveal when they choose between the actual foods. To examine this issue, we adopted a behavioral paradigm that provides a measure of the value capuchins assign to different foods. Subjects choose between two foods, one of which is preferred, offered in variable amounts. When offered the choice between a unit quantity of each food, subjects choose (by definition) the preferred food. However, if the less preferred food is offered in sufficiently large amounts, subjects will choose it. The relative value of the two foods can be inferred from the indifference point—the quantity ratio for which the subject chooses either food equally often [22]–[24]. Qualitatively, this behavioral paradigm highlights two fundamental traits of economic choice behavior. First, individuals have strict economic preferences: away from the indifference point, their choices are typically very consistent. Second, individuals' choices satisfy transitivity. In other words, if an individual is indifferent between foods X and Y and if it is indifferent between foods Y and Z, the individual is also indifferent between foods X and Z. Quantitatively, this behavioral paradigm provides an operational measure of the value individuals subjectively assign to different foods.

Transitivity is one of the main axioms of standard economic theory and is a fundamental trait of rational decision-making [25], [26]. Only a few studies examined preference transitivity in non-human primates. At the behavioral level, both capuchins and rhesus macaques (*Macaca mulatta*) combine the relative value assigned to three foods (or juices) according to transitivity [23], [24]. Furthermore, when rhesus macaques are presented with binary choices between two types of juice in variable amounts, neurons in the orbitofrontal cortex encode the value of the offered and chosen juices in a menu invariant way, suggesting that preference transitivity might be rooted in the activity of these neurons [23].

In this study, we compared preferences revealed in the real (food) and in the symbolic (token) conditions. We trained five capuchins to associate three different foods with three different types of tokens. In separate sessions, we then presented monkeys with pair-wise choices between actual foods (Food condition) or between tokens associated with the same foods (Token condition). Finally, we compared the relative values measured using tokens with those measured with the actual foods. Notably, the cognitive demands of this situation are much more challenging than in previous studies carried out in capuchins [12], [13]. Indeed, in order to choose between different quantities of tokens corresponding to qualitatively different foods, capuchins should recall the association between each token and the corresponding food, evaluate the amount of each token array, estimate the relative value of the two offers, and finally make up their mind on which option to choose. We envision three possible results. One possibility is that capuchins use the same cognitive mechanism to reason on tokens as they do with food; as a consequence, their performance will not differ in the two contexts. Alternatively, capuchins could find it more difficult to deal with tokens than with food; thus, their choice pattern will be more consistent with transitivity with food rather than with tokens. Finally, tokens may aid capuchins to override the incentive value of the immediately available food, as described for chimpanzees [9]–[11], and to achieve psychological distancing (i.e., to separate cognitively from the immediate behavioral environment, thus directing attention away from the salient features of the stimulus, 27–30). If this were the case, capuchins would deal with tokens better than with food, and therefore their choice pattern will be more consistent with transitivity with tokens rather than with food.

## RESULTS

**Figure 1:**
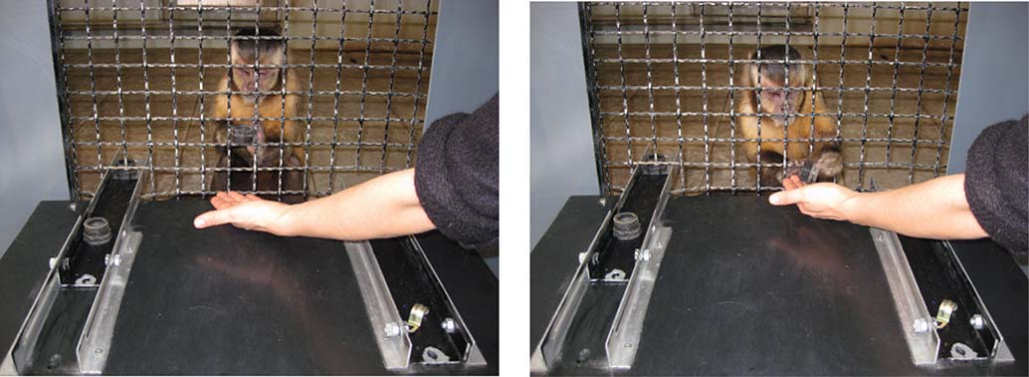
Experimental apparatus. The apparatus is positioned in front of the indoor compartment and the experimenter is nearby the apparatus, facing the subject. The subject can reach each tray through the corresponding opening in the wire mesh (8.5 cm×3.8 cm each). This photo depicts Robot, a male capuchin, who has just selected three black plastic tokens (each corresponding to one piece of dried apricot, food C) preferring them to one brass hook (corresponding to one cheerios, food A). Robot is exchanging the first plastic token for one piece of dried apricot.

Five captive-born capuchin monkeys were individually tested. Each subject was presented with a “choice apparatus”, constituted by a platform with two sliding trays where different quantities of food or tokens (according to condition) were available (Figure 1). The subject could choose one of the two offers (by pulling one of the two sliding trays) on the basis of the amount of food or tokens presented. In the preliminary phase, subjects were offered pair-wise choices between two foods (A:B and B:C) in order to select three foods such as A was preferred to B, and B was preferred to C. Then, in the Food condition, preference transitivity was tested by presenting capuchins with binary choices between different quantities of the three foods, labeled A, B and C in decreasing order of preference (Table 1, Video S1, S2, S3, S4). Subsequently, in the training phase, subjects learned to exchange three valueless tokens for the three types of food used in the Food condition (Table 2). Finally, in the Token condition, preference transitivity was tested by presenting capuchins with binary choices between different quantities of the three tokens, labeled A, B and C in decreasing order of preference (Table 1, Video S5, S6, S7, S8, S9, S10). In each trial, after choosing an offer type capuchins were required to exchange the token(s) selected (one at a time) before the experimenter administered the next trial. Each token exchange took a few seconds and it was rewarded with one piece of food.

**Table 1:** Type of trials presented in the Food condition and in the Token condition for each subject and for each item pair.

SUBJECT	ITEM PAIR	FOOD	TOKEN
	A vs. B	2A: 1B…1A: 4B	1A: 1B…1A: 6B
**Carlotta**	B vs. C	2B: 1C…1B: 4C	2B: 1C…1B: 6C
	A vs. C	2A: 1C…1A: 6C	3A: 1C…1A: 6C
	A vs. B	2A: 1B…1A: 4B	2A: 1B…1A: 5B
**Paprica**	B vs. C	2B: 1C…1B: 4C	3B: 1C…1B: 4C
	A vs. C	2A: 1C…1A: 4C	2A: 1C…1A: 6C
	A vs. B	2A: 1B…1A: 4B	2A: 1B…1A: 5B
**Robot**	B vs. C	2B: 1C…1B: 4C	3B: 1C…1B: 5C
	A vs. C	2A: 1C…1A: 6C	2A: 1C…1A: 6C
	A vs. B	2A: 1B…1A: 4B	2A: 1B…1A: 5B
**Sandokan**	B vs. C	2B: 1C…1B: 4C	2B: 1C…1B: 6C
	A vs. C	2A: 1C…1A: 6C	1A: 1C…1A: 6C
	A vs. B	2A: 1B…1A: 4B	2A: 1B…1A: 4B
**Gal**	B vs. C	2B: 1C…1B: 4C	3B: 1C…1B: 6C
	A vs. C	2A: 1C…1A: 6C	1A: 1C…1A: 6C

**Figure 2:**
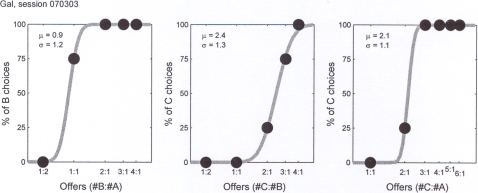
Food condition. The three panels show the choice patterns recorded for food pairs A:B, B:C and A:C, respectively. In the first panel, the x axis represents the offer type, and different offer types are ordered by the ratio of *q_B_* / *q_A_*, where *q_A_* and *q_B_* are the quantities of foods A and B offered to the subject. The y-axis represents the percentage of trials in which the subject chose item B. Analogously, in the second and third panel, the y-axis represents the percentage of trials the subject chooses food C. In this session, the subject is offered cheerios as food A, dried pineapple as food B, and rice krispies as food C. The sigmoid fits provide the relative values V(A) = 0.9 V(B), V(B) = 2.4 V(C), and V(A) = 2.2 V(C); therefore, subject's choices satisfy value transitivity since 0.9 * 2.4 ∼ 2.2.

Both in the real (food) and in the symbolic (token) conditions, the quantities of the two items offered to the monkey for any given item pair varied from trial to trial (Table 1). We thus obtained in each session three choice patterns corresponding to the three item pairs. In the Food condition, capuchins generally had strict economic preferences (i.e., for offer types away from the indifference point, data points were close to 0% or 100%). Figure 2 shows the behaviour recorded in a representative session in the Food condition. To compute relative values, we fitted each choice pattern with a “normal sigmoid”, and we interpreted the underlying Gaussian as a distribution for the relative value. The mean (*μ*) and variance (*σ* ^2^) of the distribution thus represent the estimate for the relative value and the relative error of measure (see Methods for details).

**Figure 3:**
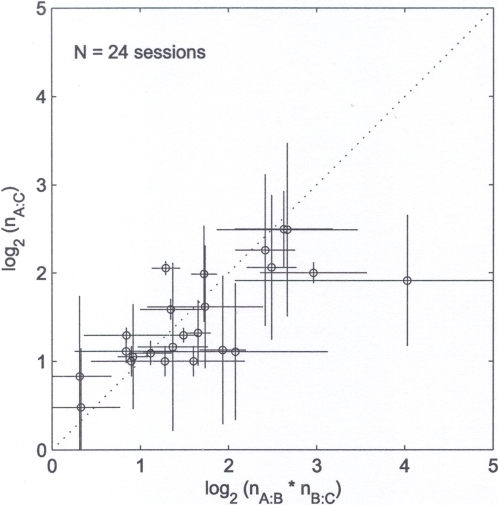
Food condition. The x-axis represents the product n_A:B_* n_B:C_, the y-axis represents n_A:C_, and each data point represents one of the 24 sessions consistent with value transitivity.

In all sessions but one, we could evaluate the relative value of each food pair (see below). As shown in Figure 3, in 96% of these sessions (23 out of 24 sessions), measured relative values satisfied value transitivity (z-test, p>0.05). This held true for all subjects, i.e. Gal (5/5 sessions), Paprica (5/5 sessions), Robot (4/5 sessions, in one session we could not evaluate the relative value of the A:C food pair), Sandokan (5/5 sessions), and Carlotta (4/5 sessions, in one session her behavior was not consistent with transitivity; p = 0.03). For all subjects but Robot, the first session of the Food condition was always consistent with transitivity.

**Figure 4:**
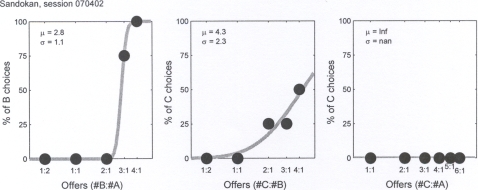
Token condition. The three panels show the choice patterns recorded for token pairs A:B, B:C and A:C. Here the subject is offered green chips as tokens A (corresponding to one cheerios each), black plastic tubes as tokens B (corresponding to one piece of dried apricot each), and brass hooks as tokens C (corresponding to one sunflower seed each). The sigmoid fits provide the relative values V(A) = 2.8 V(B) and V(B) = 4.3 V(C); however, when facing the choice between one token A and up to six tokens C, this subject always chose the single token A, thus we could not evaluate the relative value of A vs. C.

In the Token condition, in 13 out of 25 sessions we could not evaluate the relative value of at least one of the three pairs because the subject consistently chose one of the two types of token. Figure 4 shows the behavior recorded in one of the sessions in which we could not evaluate the relative value of the A:C token pair. In the remaining 12 sessions, we could evaluate the relative values of all the three token pairs and capuchins generally had strict economic preferences. As shown in Figure 4, in all these 12 sessions measured relative values satisfied transitivity (z-test, p>0.05). In particular, this held true for four out of five subjects, i.e. Gal (5/5 sessions), Paprica (4/5 sessions), Robot (2/5 sessions), and Sandokan (1/5 sessions); for all the above subjects, the first session of the Token condition was always consistent with transitivity.

To compare the results obtained in the Food condition and in the Token condition, we performed a repeated measures MANOVA (including sessions where it was possible to evaluate the relative value of each item pair, N = 24 in the Food condition and N = 12 in the Token condition) . The results showed that there was no significant relationship between the estimate of the relative value and its variability, corresponding respectively to the mean and variance of the Gaussian distribution (F_2,2_ = 11.65, p = 0.08, η^2^_p_ = 0.92). The relative value was significantly higher in the Token condition than in the Food condition (F_1,3_ = 12.35, p = 0.04, η^2^_p_ = 0.80), whereas the variability in capuchins' performance did not significantly differ across conditions (F_1,3_ = 3.51, p = 0.16, η^2^_p_ = 0.54).

## DISCUSSION

Our results can be summarized as follows. *Qualitatively*, preferences revealed by the way of tokens were similar to those measured with the actual foods. Specifically, when choosing between tokens, capuchins displayed strict economic preferences and choices satisfied transitivity since the first session for all subjects but one. These results confirm and extend previous findings obtained in non-human primates faced with choices between real foods or juices [23], [24] and with relative numerousness judgments between food or tokens [13]. *Quantitatively*, however, values measured by the way of tokens differed systematically from those measured with the actual foods. In particular, for any pair of foods, the relative value of the preferred food tended to increase when monkeys chose between the corresponding tokens. As a consequence, while in the Food condition it was generally possible to assess the relative value of the items, in about half of the sessions carried out in the Token condition this was not the case. The fact that choice patterns were otherwise qualitatively similar in the two conditions suggests that by increasing the number of the less preferred tokens (e.g., presenting more than 6 tokens C vs. 1 token A) we might induce capuchins to choose the less preferred but more numerous type of token.

Overall, these results suggest that capuchins use similar cognitive mechanisms when evaluating options in both real and symbolic contexts. Indeed, capuchins' preferences satisfied transitivity in both contexts. At the same time, tokens were not dealt with exactly as the food they stand for, since relative values were higher in the Token condition than in the Food condition. Several factors could account for this result. First, the high memory load due to recalling the association between each token and the corresponding food. However, high memory load should have led to a more “noisy” pattern of choice in the Token condition, but this did not seem to be the case, since the variability in capuchins' performance (as measured by the variance *σ* ^2^) did not significantly differ between the Food and Token conditions. Alternatively, monkeys' behavior could be explained by a decreased motivation due to the delayed feedback inherent in token exchange. This hypothesis seems, however, unlikely because our subjects never refused to participate and completed all the token trials. Moreover, the same two individuals for which tokens increased most the relative values between item pairs (Sandokan and Carlotta) were the best performers in a previous study on the estimation and combination of token quantities, where temporal discounting was at stake [12]. Nonetheless, since each token exchange for the corresponding food takes a few seconds, we cannot rule out that capuchins may have discounted the offers involving a higher number of tokens [31].

Finally, the different results obtained in the Token condition compared to the Food condition could reflect a difficulty to achieve a dual representation [3]. Specifically, capuchins may find it difficult to grasp the dual nature of tokens as it is the case for young children. In humans, the understanding and use of symbolic artifacts develops slowly because simultaneously representing both the concrete object itself and its abstract relation to what it stands for is complex [5]. DeLoache and colleagues have extensively investigated how young children use scale models (i.e., realistic miniature models of a familiar playroom) as a source of information for solving a retrieval problem. In a typical trial, children observed an experimenter hide a miniature toy in the model and were then asked to find the larger toy in the analogous location of the playroom. Children understanding of the model-playroom relation and the ability to successfully find the corresponding larger toy in the playroom developed between 2.5 and 3.0 years of age [3]–[5]. In chimpanzees tested in a similar version of this task, only a few individuals could inhibit perseverative object-oriented responses and successfully retrieved the hidden item [11].

Young children seem to fail in the scale model task because they are attentive to the real object rather than to what it stands for. When the salience of the model is decreased (for example substituting the scale model with a video clip), or when there is no need for dual representation (children are told that a “shrinking machine” transformed the playroom into the miniature model), performance improves. In contrast, performance declines when the physical salience of the scale model is increased by allowing children to play with the model before performing the task [3], [5]. A similar phenomenon might explain our results. In the Token condition capuchins might have focused on the quality of the preferred token disregarding the quantities of the two alternatives, thus choosing this token more often than the corresponding food in the Food condition. Future studies should assess whether preventing the physical interaction with tokens by eliminating the exchange procedure modifies capuchins' performance.

Interestingly, in humans a complete appreciation of the symbolic nature of tokens took long time to be achieved [32]. Around 8500 years B.C. the Sumerians started keeping track of trades by employing a system based on small clay tokens shaped differently depending on the good they stood for. The evolution of this token system reflected Sumerians' socioeconomic development. As their trades expanded, Sumerians needed to transport tokens in clay pots and, to readily identify the content of each pot, they engraved on their surfaces the type and number of the tokens contained. Nonetheless, Sumerians did not immediately realized how abstract this symbolic system could be and only after several millennia both number representation and writing evolved from the engraved clay pots.

Finally, the increased abstraction of the Token condition did not ameliorate capuchins' performance, as reported for chimpanzees [9]–[11] and young children [30], [33], [34] when symbolic representations substituted for real food. Again, limited mastery of the dual nature of tokens may have prevented capuchins from achieving psychological distancing [27]–[30]; however, this hypothesis requires further studies examining whether tokens enhance performance in tasks where inhibition is critical for success.

In conclusion, capuchin monkeys' behavior with tokens is not simply the product of instrumental conditioning, though tokens have not gained yet the status of human money. Our findings suggest that capuchins indeed treat tokens as symbols, despite experiencing the cognitive burdens imposed by symbolic representation. Thus, also non-apes have undertaken the path of symbol use and understanding, though they are far from achieving full symbolic competence.

## MATERIALS AND METHODS

### Subjects and apparatus

Five captive-born capuchin monkeys (three males, two females, average 15.4 years, range 7–23) were tested. All subjects were already proficient in token exchange and had experience in cognitive and number-related tasks [12], [13]. No subject but one (Robot) had already participated in a previous study on transitivity of food preferences [24].

They lived in three groups at the Primate Center of the Institute of Cognitive Sciences and Technologies of CNR, Rome; each group was housed in indoor–outdoor compartments (total area: 65.4–139.5 m^3^, depending on group size) and tested in one of the two indoor compartments (12.2 m^3^ each, for all groups). All compartments were furnished with wooden perches, tree trunks and branches. Separation for individual testing was achieved by splitting the group into smaller units by means of sliding doors and then allowing one individual to enter the indoor compartment. This procedure was part of the daily routine. Monkeys were not food deprived for testing. The main meal took place in the afternoon when fresh fruits, vegetables and monkey chow were provided. Water was available *ad libitum*. This study complied with protocols approved by the Italian Health Ministry and all procedures were performed in full accordance with the European law on humane care and use of laboratory animals.

Subjects were tested individually in the indoor compartment; the apparatus was a black plastic table (65 cm×64 cm×13.5 cm) with two sliding aluminum trays (6.5×40 cm; 2.5 cm high), positioned at 32 cm distance from one another. Each tray had two holes (1.4 cm in diameter), one at each end; one served to allow the subject's pulling, whereas the other hole allowed the experimenter to block the tray by inserting a pin into it. All subjects were already familiar with the apparatus [12], [13]. The experiment proceeded in phases. In the preliminary phase, subjects were tested for their binary food preferences. Subsequently, preference transitivity was tested in choices between foods (Food condition). Then, in the training phase, subjects learned to exchange tokens for food. Finally, preference transitivity was tested in choices between tokens (Token condition).

### Preliminary phase

Capuchins' preference for two pairs of foods (A:B and B:C, see below) was assessed. First, we carried out the preference test for the pair A:B, and then for the pair B:C. In each session, capuchins faced binary choices between different quantities of a pair (A:B or B:C). Before the beginning of each session, eight familiarization trials were carried out by presenting pair-wise comparisons between the two foods (one piece each). According to subjects' preferences, foods were referred to as food A (high-preferred) and food B (low-preferred). Similarly, when testing the other food pair, foods were referred to as food B (high-preferred) and food C (low-preferred). Individual preferences varied so that labels A, B, and C referred to different foods for different subjects (Table 2).

**Table 2:** Food and token triads for each subject.

SUBJECT	ITEM	FOOD	TOKEN
	A	cheerios	green chip
**Carlotta**	B	parmesan	black plastic tube
	C	sunflower seed	brass hook
	A	pistachio	black plastic tube
**Paprica**	B	dried pineapple	brass hook
	C	sunflower seed	green chip
	A	cheerios	brass hook
**Robot**	B	black olives	green chip
	C	dried apricot	black plastic tube
	A	cheerios	green chip
**Sandokan**	B	dried apricot	black plastic tube
	C	sunflower seed	brass hook
	A	cheerios	brass hook
**Gal**	B	dried pineapple	green chip
	C	rice krispies	black plastic tube

A items are preferred to B items, and B items are preferred to C items.

For the A:B pair, in each trial capuchins faced a binary choice between one or two pieces of food A and one to five pieces of food B. Therefore, the following comparisons were presented: 2A:1B, 1A:1B, 1A:2B, 1A:3B, 1A:4B, and 1A:5B; each combination was presented eight times for a total of 48 trials in a pseudo-random order. The left/right arrangement of the offers was counterbalanced within each session. Each subject received one session a day for a total of five sessions. The same procedure was employed for the B:C pair.

Testing was carried out by two experimenters: experimenter 1 sat in front of the subject's indoor compartment, with the apparatus placed on the floor between the experimenter and the capuchins' compartment. Placed between the experimenters were two opaque containers, each containing pieces of one type of food. Experimenter 2 sat next to experimenter 1 and during baiting she covered the apparatus with an opaque screen to prevent the subject from seeing the process. Then, experimenter 2 lifted the opaque screen and experimenter 1 pushed the apparatus towards the wire mesh, so that the monkey could pull one of the two trays. Both experimenters refrained from looking at the apparatus so as not to provide cues to the subject. The inter-trial interval was about 10 s.

### Food condition

In each session, capuchins faced choices between different quantities of three foods, labeled A, B and C in decreasing order of preference. Choices were binary and trials with the three pairs of foods (A:B, B:C and C-A) were interleaved pseudo-randomly. Before the beginning of each session, nine familiarization trials were carried out by presenting for three times all the possible pair-wise comparisons between the three foods (one piece of food of each type). Foods were referred to as food A (high-preferred), food B (medium-preferred), and food C (low-preferred).

In each trial capuchins could face a binary choice between: (1) one or two pieces of food A and one to four pieces of food B, (2) one or two pieces of food B and one to four pieces of food C, and (3) one or two pieces of food A and one to six pieces of food C. In a session, each comparison was presented four times. For each food pair, according to each individual's indifference point, the type and number of trials presented varied across sessions, from a minimum of 60 to a maximum of 64 trials per session; no subject received all the possible 72 comparisons within a session (Table 1). The left/right arrangement of the offers was counterbalanced within each session. Each subject received one session a day for a total of five sessions. All the other features of the procedure were the same as in the preliminary phase.

### Token condition

The same five capuchins were presented with the Token condition after completing all the five sessions in the Food condition. We used the same subjects and apparatus as in the Food condition.

#### (a) Tokens

Tokens were objects of similar dimensions, differing in shape, material and color; in particular, we used green chips, black plastic tubes, and brass hooks. These objects were familiar to the subjects but never used in previous studies. The three tokens were pseudo-randomly assigned to the three types of food across subjects (Table 2).

#### (b) Training

Subjects learned to associate each type of token to one of the three foods used in the Food condition (Table 2). Therefore, token A was associated with the high-preferred food, token B with the medium-preferred food, and token C with the low-preferred food. The training procedure consisted of placing 12 tokens of the same type (i.e., associated to the same type of food) into the indoor compartment, and repeatedly saying ‘give me’ to the monkey while requesting a token, with left hand outstretched and palm up. The reward was given upon the placement of one token into the experimenter's left hand. There was a 10-s interval between one trial and the next one. Incorrect exchanges, in which tokens were thrown or incorrectly placed into the experimenter's hand, were not rewarded. Moreover, when the subject did not exchange a token within 30 s, the trial was considered incorrect and a new trial started after 10 s.

Subjects received a training session per day. Each session consisted of two blocks of 12 trials each, for a total of 24 trials. Criterion was set at 90% correct responses within two consecutive sessions. Each subject was trained to exchange one type of token (A, B, or C) at a time, and the order in which they learned to exchange the three tokens was randomly determined. When criterion was reached for all types of token, subjects received nine sessions of consolidation with tokens A, B and C alternated across days. Capuchins completed training (including the nine sessions of consolidation) in an average of 18.2±1.7 sessions. In particular, they reached criterion in an average of 2.8±0.4 sessions for token A (range: 2–4), 2.8±0.8 sessions for token B (range: 2–6), and 3.4±1.4 sessions for token C (range: 2–9). The rate of training of the present study is similar to that reported for capuchins learning to associate different type of tokens with different quantities of food [12], [13].

#### (c) Procedure

In each session, capuchins faced binary choices between different quantities of three tokens (A:B, B:C, and A:C). Before the beginning of each session, nine familiarization trials were carried out by presenting for three times all the possible pair-wise comparisons between the three tokens (one token of each type). According to subjects' preferences, tokens were referred to as token A (associated to the high-preferred food), token B (associated to the medium-preferred food), and token C (associated to the low-preferred food).

In each trial capuchins could face a binary choice between: (1) one or two tokens A and one to six tokens B, (2) one to three token(s) B and one to six token(s) C, and (3) one or two token(s) A and one to six token(s) C. In each trial, after choosing an offer type capuchins were required to exchange the token(s) selected (one at a time) before the experimenter administered the next trial; each token exchange took a few seconds and was rewarded with one piece of food (Table 2). Typically, capuchins exchanged correctly, and in the very few cases in which they did not do so, the experimenter gave the token back to the subject so that s/he could exchange it again.

In a session, each comparison was presented four times. For each token pair, according to each individual's indifference point, the type and number of trials presented varied across sessions, from a minimum of 60 to a maximum of 76 trials per session in a pseudo-random order; no subject received all the possible 88 comparisons within a session (Table 1). The left/right arrangement of the offers was counterbalanced within each session. Each subject received one session a day for a total of five sessions. All the other features of the procedure were the same as in the Food condition. The study took place between February and May 2007.

### Analysis of choice patterns

We analyzed choice patterns using the method employed by Padoa-Schioppa & Assad [22], [23]. We refer to “relative” values because behavioral analyses allow measuring quantities of different goods on a common value scale up to a scaling factor. Our measure of relative value rests on the assumption of linear indifference curves: if a monkey is repeatedly offered the choice between quantities *qX* and *qY* of items X and Y (offer *q_Y_*Y : *q_X_*X), the rate of “Y” choices only depends on the ratio *q_Y_* / *q_X_* [23].

Choice patterns recorded for each pair of items X and Y are expressed as a function of log(*q_Y_* / *q_X_*), where *q_X_* and *q_Y_* are, respectively, the quantities of items X and Y offered to the monkey. For each item pair, we then fit the percentage of “Y” choices with a normal sigmoid, which is a normal cumulative distribution function of the form S(x) = ∫^x^_−∞_ = N (*t, μ, σ*) *dt*. We interpret the underlying Gaussian (which has mean *μ* and variance *σ* ^2^) as a probability distribution for the log relative value, and we compute the estimated relative value of the two items n = exp(μ). The relative value corresponds to the indifference point, i.e. the ratio of quantities for which the monkey would choose either item equally often. We indicate with V(X) the value of X, and with n_X:Y_ the relative value of items X and Y, such that V(X) = n_X:Y_ V(Y). For each session, we thus obtain the three probability distributions for the log relative values u = log(n_A:B_), v = log(n_B:C_) and w = log(n_A:C_). Under the assumption of linear indifference curves, indifference transitivity is satisfied if the following relationship holds statistically true: n_A:B_ * n_B:C_ = n_A:C_. We refer to this condition as “value transitivity.” Testing whether values satisfy transitivity reduces to testing whether the identity u+v = w holds statistically true. Because u, v and w are all normally distributed variables, transitivity violations can be identified with a z-test [23].

## Supporting Information

Video S1 Food condition. Carlotta, a female capuchin, has a choice between one cheerios (food A, on the left) and three pieces of parmesan cheese (food B, on the right). She selects the three pieces of parmesan cheese by pulling the corresponding tray.
(4.79 MB MPG)

Video S2 Food condition. Carlotta has a choice between one cheerios (food A, on the left) and three sunflower seeds (food C, on the right). She selects the single cheerios by pulling the corresponding tray.
(4.78 MB MPG)

Video S3 Food condition. Carlotta has a choice between three sunflower seeds (food C, on the left) and one piece of parmesan cheese (food B, on the right). She selects the three sunflower seeds by pulling the corresponding tray.
(4.56 MB MPG)

Video S4 Food condition. Carlotta has a choice between five sunflower seeds (food C, on the left) and one cheerios (food A, on the right). She selects the five sunflower seeds by pulling the corresponding tray.
(5.82 MB MPG)

Video S5 Token condition. Carlotta has a choice between one green poker chip (token A, corresponding to one cheerios, on the left) and three brass hooks (token C, corresponding to one sunflower seed each, on the right). She selects the token A by pulling the corresponding tray.
(5.28 MB MPG)

Video S6 Token condition. Carlotta has a choice between three black plastic tubes (token B, corresponding to one piece of parmesan cheese each, on the left) and one green poker chip (token A, corresponding to one cheerios, on the right). She selects the three tokens B by pulling the corresponding tray.
(9.35 MB MPG)

Video S7 Token condition. Carlotta has a choice between one black plastic tube (token B, corresponding to one piece of parmesan cheese, on the left) and three brass hooks (token C, corresponding to one sunflower seed each, on the right). She selects the single token B by pulling the corresponding tray.
(6.34 MB MPG)

Video S8 Token condition. Carlotta has a choice between one black plastic tube (token B, corresponding to one piece of parmesan cheese, on the left) and four brass hooks (token C, corresponding to one sunflower seed each, on the right). She selects the four tokens C by pulling the corresponding tray.
(10.39 MB MPG)

Video S9 Token condition. Carlotta has a choice between one green poker chip (token A, corresponding to one cheerios, on the left) and five brass hooks (token C, corresponding to one sunflower seed each, on the right). She selects the single token A by pulling the corresponding tray.
(5.53 MB MPG)

Video S10 Token condition. Carlotta has a choice between six brass hooks (token C, corresponding to one sunflower seed each, on the left) and one green poker chip (token A, corresponding to one cheerios, on the right). She selects the six tokens C by pulling the corresponding tray.
(12.60 MB MPG)
